# Carotenoid-Rich Brain Nutrient Pattern Is Positively Correlated With Higher Cognition and Lower Depression in the Oldest Old With No Dementia

**DOI:** 10.3389/fnut.2021.704691

**Published:** 2021-06-29

**Authors:** Jirayu Tanprasertsuk, Tammy M. Scott, Aron K. Barbey, Kathryn Barger, Xiang-Dong Wang, Mary Ann Johnson, Leonard W. Poon, Rohini Vishwanathan, Nirupa R. Matthan, Alice H. Lichtenstein, Guylaine Ferland, Elizabeth J. Johnson

**Affiliations:** ^1^Gerald J. and Dorothy R. Friedman School of Nutrition Science and Policy, Tufts University, Boston, MA, United States; ^2^Department of Psychology, Beckman Institute for Advanced Science and Technology, University of Illinois at Urbana-Champaign, Urbana, IL, United States; ^3^Jean Mayer USDA Human Nutrition Research Center on Aging at Tufts University, Boston, MA, United States; ^4^Department of Nutrition and Health Sciences, University of Nebraska Lincoln, Lincoln, NE, United States; ^5^Institute of Gerontology, College of Public Health, University of Georgia, Athens, GA, United States; ^6^Département de Nutrition, Université de Montréal, Montréal, QC, Canada

**Keywords:** dementia, cognition, carotenoids, vitamin A, vitamin E, vitamin K, fatty acids, centenarian adults

## Abstract

**Background:** Healthy dietary patterns are related to better cognitive health in aging populations. While levels of individual nutrients in neural tissues are individually associated with cognitive function, the investigation of nutrient patterns in human brain tissue has not been conducted.

**Methods:** Brain tissues were acquired from frontal and temporal cortices of 47 centenarians from the Georgia Centenarian Study. Fat-soluble nutrients (carotenoids, vitamins A, E, K, and fatty acids [FA]) were measured and averaged from the two brain regions. Nutrient patterns were constructed using principal component analysis. Cognitive composite scores were constructed from cognitive assessment from the time point closest to death. Dementia status was rated by Global Deterioration Scale (GDS). Pearson's correlation coefficients between NP scores and cognitive composite scores were calculated controlling for sex, education, hypertension, diabetes, and *APOE* ε4 allele.

**Result:** Among non-demented subjects (GDS = 1–3, *n* = 23), a nutrient pattern higher in carotenoids was consistently associated with better performance on global cognition (*r* = 0.38, *p* = 0.070), memory (*r* = 0.38, *p* = 0.073), language (*r* = 0.42, *p* = 0.046), and lower depression (*r* = −0.40, *p* = 0.090). The findings were confirmed with univariate analysis.

**Conclusion:** Both multivariate and univariate analyses demonstrate that brain nutrient pattern explained mainly by carotenoid concentrations is correlated with cognitive function among subjects who had no dementia. Investigation of their synergistic roles on the prevention of age-related cognitive impairment remains to be performed.

## Introduction

Advancing age is the number one risk factor of age-related cognitive impairment and dementia, representing a major public health epidemic among older Americans (1). A systematic review of cross-sectional studies and longitudinal cohorts has identified a relation between diet quality and cognitive health in the aging population (2). A healthy diet covers a variety of dietary patterns such as Mediterranean diet (MeDi) (3, 4), Healthy Dietary Index (HDI) diet (5), Healthy Eating Index (HEI) diet (6), Dietary Approaches to Stop Hypertension (DASH) diet (7), and Mediterranean-DASH Intervention for Neurodegenerative Delay (MIND) diet (8). In some studies, dietary patterns are derived *a posteriori* with cluster analysis, factor analysis (e.g., principal component analysis [PCA]), or reduced rank regression (9–13). Though healthy dietary patterns have been diversely defined, they share common components of high intake of fruits, vegetables, whole grains, nuts, seeds, fish, and limited intake of added sugar, sodium, high-fat dairy products, red, and processed meat (2).

Traditionally, habitual dietary intake has been estimated using subjective recall data derived from food frequency questionnaires (FFQ). This approach, despite its direct translation for establishing dietary recommendations, does not account for recall bias, particularly among subjects with varied cognitive performance, and inter-individual variability in the nutrient absorption and metabolism (14). There has been an attempt to overcome these issues by constructing serum nutrient patterns (NP) as a marker of intake among subjects in the Oregon Brain Aging Study (15), and the Illinois Brain Aging Study (16–18). However, given that cognitive processes originate from the human brain, particularly in neocortices, and that nutrient uptake into the central nervous system (CNS) is strictly regulated at the blood-brain barrier (BBB) (19), we need to expand the scope of investigation further into the CNS for a greater apprehension of nutrition's roles in the aging brain.

While the evidence for relations between diet quality and cognitive function has been largely consistent among observational studies, the evidence from clinical trials of dietary supplements has been mixed (20). When investigating the relationship between nutrition and age-related diseases, the importance of examining nutrition as dietary patterns or NPs have been highlighted (21, 22), as most individuals acquire nutrients predominantly from foods, rather than supplements, throughout their lifespan. From a biochemical and molecular perspective, the etiology of age-related dementia, despite its heterogeneity, shares multiple mechanisms including cardiometabolic risk factors, elevated oxidative stress, neuroinflammation, and impaired AMP-activated kinase signaling (23). All of these factors can potentially be regulated by multiple dietary components. The pathology of age-related cognitive impairment is also different from cognitive symptoms caused by a deficiency of a single nutrient that may manifest during a shorter period of time and may be reversible—such as dementia caused by vitamin B12 or niacin deficiency, and Wernicke-Korsakoff syndrome caused by a genetic predisposition “to thiamin” deficiency (24, 25).

Therefore, the present research study was proposed based on the rationale that many fat-soluble nutrients (carotenoids, vitamins A, E, K, and fatty acids [FA]) are present in human brain (26–31), and that they are a part of dietary patterns and serum NPs previously reported to be associated with better cognitive function in multiple aging cohorts (2, 15, 26, 28, 32, 33). This was accomplished by constructing *a posteriori* NPs of fat-soluble nutrients measured in brain tissues acquired post-mortem from a subset of centenarians (defined as ≥98 years) who were enrolled in the Georgia Centenarian Study (GCS)—the longest running centenarian study in the U.S. to date (34, 35). Subsequently, the relationship between constructed NPs and cognitive performance at the time point closest to death was cross-sectionally investigated. Findings from this novel study may also provide insights into the role of nutrition in cognition in the oldest old, which may be similar or different from lesser aged older adults.

## Materials and Methods

### Subject Recruitment and Brain Collection

The design of GCS, objectives, protocols of subject recruitment, and brain collection have been previously described in detail (34, 35). Briefly, the GCS was a population-based study conducted in 44 counties in northern Georgia. The GCS was primarily designed to identify biological, psychological, and social factors contributing to survivorship and successful aging. Brain tissues from frontal (FC) and temporal cortices (TC) were collected from 47 subjects who were a subset of centenarians enrolled in the phase III of the GCS (2001–2007) and gave consent to donate brain tissue upon death. After tissue collection, all samples were coded and stored at −80°C until the measures of nutrient concentration. All protocols were performed with an approval from the University of Georgia Institutional Review Board. Separate approval for using de-identified data for the present analyses was obtained from the Tufts University/Tufts Medical Center Institutional Review Board.

### Nutrient Concentration Measures in Brain Tissues

Protocols for brain lipid extraction, separation, quantification, and concentrations have been previously and separately described for carotenoids, vitamin A (retinol), vitamin E (α- and γ-tocopherol [TP]), vitamin K (phylloquinone [PK] and menaquinone-4 [MK-4]), and individual FAs (27, 28, 36–39). In short, separation and quantification of five major dietary carotenoids (lutein, zeaxanthin, cryptoxanthin, β-carotene, and lycopene), retinol, and TPs were performed using high-performance liquid chromatography (HPLC) coupled with a photodiode array detector. The limit of detection (LOD) was 0.2 pmol for carotenoids, 2.0 pmol for retinol, 2.7 pmol for TPs per injection. Only levels of the all-*trans* isomer of each carotenoid, which is the most predominant isomer in human brain tissues, are reported (26, 40). Separation and detection of PK and MK-4 were performed using HPLC coupled with a fluorescence detector. The LOD was 0.03 pmol for both vitamin K vitamers. Separation and detection of individual FAs were performed using a gas chromatography coupled with a flame ionization detector, and expressed as molar percentage (mol%). Total saturated FAs (SFAs) represent the sum of 10:0, 12:0, 14:0, 15:0, 16:0, 18:0, 20:0, 22:0, and 24:0. Total monounsaturated FAs (MUFAs) represent the sum of 16:1 (n-9), 16:1 (n-7), 18:1 (n-9), 18:1 (n-7), 20:1 (n-9), 22:1 (n-9), and 24:1 (n-9). Total omega-3 polyunsaturated FAs (n-3 PUFAs) represent the sum of 18:3, 18:4, 20:3, 20:5, 22:5, and 22:6. Total omega-6 polyunsaturated FAs (n-6 PUFAs) represent the sum of 18:2, 18:3, 20:2, 20:3, 20:4, 22:2, 22:4, and 22:5. Total *trans*-FAs represent the sum of 16:1 (n-9), 16:1 (n-7), trans-6-octadecenoic acid (18:1, n-10 to 12), 18:1 (n-9), 18:1 (n-7), *trans*-9, *trans*-12- octadecenoic acid (18:2 TT/TCTX), and conjugated linoleic acid (18:2, CLA).

### Cognitive Assessment and Cognitive Domain Composite Scores

After enrollment in the GCS, cognitive assessment was performed every 6 months at the subject's residence as reported earlier (34). Cognitive data were obtained from the visit closest to death (<1 year for all subjects). Dementia status was assessed by geriatric psychiatrists using Global Deterioration Scale (GDS) and subjects were grouped based on GDS score. A score of 1–2 on GDS was clinically defined as no dementia; a score of 3 represented mild cognitive impairment; and a score of 4–7 represented increasing severity of dementia from mild to severe (41). Cognitive tests included Mini-Mental State Examination (MMSE, 24–30 = normal cognition; 19–23 = mild; 10–18 = moderate; or ≤ 9 = severe cognitive impairment) (42), Severe Impairment Battery (SIB, < 63 = very severely impaired cognition) (43), Fuld Object Memory Evaluation (FOME) (44), Controlled Oral Word Association Test (COWAT) (45), Wechsler Adult Intelligence Scale Third Edition (WAIS-III) Similarities (46), Behavioral Dyscontrol Scale (BDS) (47), and the Consortium to Establish a Registry for Alzheimer's Disease (CERAD) battery which included Verbal Fluency (VF), Boston Naming Test (BNT), Constructional Praxis (CP), and Word List Memory Test (WLMT) (48, 49). Depression was assessed using Geriatric Depression Scale Short Form (GDSSF) (50), and activities of daily living were assessed using Direct Assessment of Function Status (DAFS) (51). All subtests have been validated and are considered reliable measures of cognition in normal aging and in AD (52).

To calculate cognitive domain composite scores, scores from each cognitive test were normalized using z-scoring as previously reported (53). Composite scores of five cognitive domains (memory, executive function, language, visuospatial function, attention), depression, and activities of daily living were then calculated by averaging the z-scores of tests based on the method adapted from Bowman et al. (15). The calculation method has also previously been reported and shown in Supplementary Table 1. Global cognition composite scores were also derived by combining total cognitive testing z-scores, MMSE, and SIB. Missing test scores were excluded and the denominator changed accordingly for the calculation of composite scores.

### Statistical Analysis

Values are presented as mean (SD). All statistical tests were performed in R 3.5.1 with a significance level set at α = 0.05. Findings with *p* < 0.1 but > 0.05 were reported as borderline significant. Comparisons of subject characteristics between non-demented (GDS 1–3, *n* = 23) and demented subjects (GDS 4–7, *n* = 24) were performed using Student's two-sample *t*-test and Fisher's exact test for continuous and categorical variables, respectively.

NPs were derived from concentrations of carotenoids, vitamins A, E, K, SFAs, MUFAs, n-3 PUFAs, n-6 PUFAs, and *trans*-FAs averaged from FC and TC (vitamin K only from FC due to limited brain sample availability) using PCA with a function pca in the R package “pcaMethods” (54). Concentrations of all nutrients were log transformed prior to PCA. Nutrient concentration matrix was unit-variance scaled and centered. We chose non-linear iterative partial least squares algorithm to calculate principal components, or NPs in our case, which is an iterative approach for estimating independent principal components by extracting them one at a time (55). This variation of PCA can handle small amount of missing values, which in our case were PK and MK-4 concentrations from two individuals due to insufficient brain tissues for vitamin K analysis. Only NPs with eigenvalue >1 were reported.

To investigate the relationship between brain nutrient concentrations or NPs and cognitive domain composite scores, Pearson's correlation test was performed with an adjustment for covariates sex, education, hypertension, diabetes, and presence of *APOE* **ε**4 allele. Additional adjustment for antithrombotic use was performed for vitamin K, and antidepressant use for depression score. Sub-analyses in non-demented (GDS 1–3) were also performed. Heat maps aided the visualization of correlations.

## Results

### Subject Characteristics

Characteristics of all 47 subjects are reported in Table 1. By design, all subjects were ≥98 years old with an average age at death of 102.2 (2.5) years old. Eighty-nine percent were Caucasian and 89% were female. Subjects who did not finish high school accounted for 51%. Seventy percent were institutionalized at the visit closest to death. Body mass index (BMI) was 22.1 (3.9) kg/m^2^ on average, excluding one double amputee whose BMI could not be calculated. Approximately half of the subjects had hypertension (53%) while only 3 subjects had diabetes (6%). In terms of medication and supplement uses, while no subjects took cholesterol-lowering medication, the majority of subjects used at least one form of dietary supplements (72%). Twelve subjects, ten of whom had dementia, could not provide data on history of smoking and alcohol use through recall. Among those with available data, 86% never smoked and 60% never used alcohol. Only one subject was an active smoker at death.

**Table 1:** Subject characteristics.

**Characteristic**	**All subjects (*n* = 47)**	**GDS 1-3 (*n* = 23)**	**GDS 4–7 (*n* = 24)**	***P*-value^**a**^**
Age in years, mean (SD)	102.2 (2.5)	102.2 (2.3)	102.2 (2.8)	0.946
Female, *n* (%)	42 (89%)	19 (83%)	23 (96%)	0.188
Race, *n* (%)				0.348
Caucasian	42 (89%)	22 (96%)	20 (83%)	
Black	5 (11%)	1 (4%)	4 (17%)	
BMI in kg/m^2^, mean (SD)^b^	22.1 (3.9)	23.1 (3.5)	21.0 (4.0)	0.067
Hypertension, *n* (%)	25 (53%)	12 (52%)	13 (54%)	1
Diabetes, *n* (%)	3 (6%)	2 (9%)	1 (4%)	0.609
Education, *n* (%)				0.204
< High school	23 (51%)	9 (39%)	14 (64%)	
High school	12 (27%)	7 (30%)	5 (23%)	
> High school	10 (22%)	7 (30%)	3 (14%)	
No data	2	0	2	
Living, *n* (%)				0.212
Community dwelling	14 (30%)	9 (39%)	5 (21%)	
Institutionalized	33 (70%)	14 (61%)	19 (79%)	
Dietary supplement use, *n* (%)	34 (72%)	14 (61%)	20 (83%)	0.111
Medications, *n* (%)				
Antidepressants	14 (30%)	4 (17%)	10 (42%)	0.111
Antipsychotics	5 (12%)	1 (4%)	4 (17%)	0.348
Anti-inflammatory medications	5 (12%)	4 (17%)	1 (4%)	0.188
Antithrombotics	10 (21%)	5 (22%)	5 (21%)	1
Antibiotics	7 (15%)	2 (9%)	5 (21%)	0.416
Smoking, *n* (%)				0.570
Never	30 (86%)	18 (86%)	12 (86%)	
Past	4 (11%)	3 (14%)	1 (7%)	
Present	1 (3%)	0 (0%)	1 (7%)	
No data	12	2	10	
Alcohol use, *n* (%)				0.041
Never	21 (60%)	9 (43%)	12 (86%)	
Past	6 (17%)	5 (24%)	1 (7%)	
Present	8 (23%)	7 (33%)	1 (7%)	
No data	12	2	10	
*APOE*, n (%)^c^				
ε2 allele carrier	8 (17%)	3 (13%)	5 (21%)	0.701
ε4 allele carrier	8 (17%)	3 (13%)	5 (21%)	0.701

a *Comparisons between subjects whose Global Deterioration Scale (GDS) = 1–3 (non-demented) and GDS = 4–7 (demented) using Student's t-test for continuous variables and Fisher's exact test for categorical variables*.

b *Body mass index (BMI) cannot be calculated for a double amputee whose GDS = 5*.

c *All subjects who carried an ε2 or ε4 allele were ε2/ε3 or ε3/ε4 except one subject with GDS = 4 who was an ε2/ε4*.

Subject characteristics between non-demented (*n* = 23) and demented (*n* = 24) subjects, as assessed by the GDS, were not statistically different, except that BMI in non-demented subjects was marginally higher than that in demented subjects [23.1 (3.5) vs. 21.0 (3.5) kg/m^2^, *p* = 0.067] (Table 1). Although higher proportion of subjects without dementia reported a history of alcohol use (*p* = 0.041), the data were only available for only 58% of demented participants.

### Nutrient Concentrations in FC and TC, and Establishing NPs

Concentrations of nutrients averaged from the FC and TC are reported in Table 2 (vitamin K only from FC). These data have also been previously reported separately for FC and TC (27, 31, 40). Lutein was the most predominant carotenoid in all FC and TC tissues with a concentration of 79.50 (52.57) pmol/g. On the contrary, lycopene, at an average concentration of 20.41 (21.38) pmol/g, was not detected in both FC and TC in 20 subjects (43% of all subjects). Retinol, which included both free retinol, retinal, and retinyl esters before hydrolyzation during lipid extraction, had a concentration of 691.97 (305.56) pmol/g. Concentrations of α-TP and γ-TP were 66,917 (13,676) and 1,742 (1,018) pmol/g, respectively. While MK-4 was detectable in all brain tissues at 4.96 (2.32) pmol/g, PK was not detected in 17 subjects (38%). The predominant class of FA in the brain samples was SFA, which accounted for 15.36 (2.30) nmol/mg or 47.93 (1.68) mol%. Concentrations of individual FAs are shown in Supplementary Table 2. Docosahexaenoic acid (DHA, 22:6 n-3) and arachidonic acid (AA, 20:4 n-6) were the most predominant n-3 PUFA (11.90 (1.60) mol% or 90.22% of total n-3 PUFAs) and n-6 PUFA (8.54 (0.57) mol% or 66.36% of total n-6 PUFAs), respectively, in all brain tissues. Concentrations of each nutrient were not significantly different among demented and demented subjects. However, total *trans*-FA concentration was significantly higher among demented subjects (*p* = 0.020).

**Table 2:** Mean (SD) of nutrient concentrations averaged from the frontal and temporal cortices (vitamin K only in the frontal cortex).

**Nutrient**	**All subjects (*n* = 47)**	**GDS 1–3 (*n* = 23)**	**GDS 4-7 (*n* = 24)**	***P*-value^a^**
**Carotenoids (pmol/g)**				
Lutein	79.50 (52.57)	76.34 (43.72)	82.52 (60.65)	0.828
Zeaxanthin	26.97 (12.73)	28.36 (14.06)	25.63 (11.44)	0.404
Cryptoxanthin (α+β)	62.06 (67.97)	57.15 (47.61)	66.76 (83.81)	0.787
β-Carotene	55.86 (35.56)	46.22 (24.20)	65.09 (42.27)	0.130
Lycopene	20.41 (21.38)	21.63 (21.57)	19.25 (21.59)	0.901
Retinol (pmol/g)	691.97 (305.56)	674.11 (286.94)	709.10 (327.65)	0.688
**Vitamin E (pmol/g)**				
α-Tocopherol	66,917 (13,676)	68,303 (13,732)	65,588 (13,782)	0.526
γ-Tocopherol	1,742 (1,018)	1,891 (1,049)	1,600 (989)	0.208
**Vitamin K (pmol/g)**^**b**^				
Phylloquinone	0.40 (0.39)	0.35 (0.42)	0.45 (0.36)	0.276
Menaquinone-4	4.96 (2.32)	5.05 (2.82)	4.88 (1.82)	0.772
**Fatty acid (nmol/mg)**				
Total SFA	15.36 (2.30)	15.04 (2.40)	15.66 (2.21)	0.333
Total MUFA	7.00 (2.41)	6.83 (1.93)	7.17 (2.83)	0.723
Total n-3 PUFA	4.20 (0.67)	4.11 (0.73)	4.29 (0.60)	0.321
Total n-6 PUFA	5.53 (1.26)	5.41 (1.04)	5.63 (1.45)	0.569
Total *trans*-FA	0.25 (0.09)	0.22 (0.06)	0.28 (0.10)	0.020

a *Comparisons between Global Deterioration Scale (GDS) 1–3 and GDS 4–7 using Student's t-test. Log (x+1) transformation was applied prior to comparisons*.

b *Brain tissue from two subjects was not available for vitamin K measures*.

*SFA, saturated fatty acid; MUFA, monounsaturated fatty acid; PUFA, polyunsaturated fatty acid; trans-FA, trans-fatty acid*.

**Figure 1:**
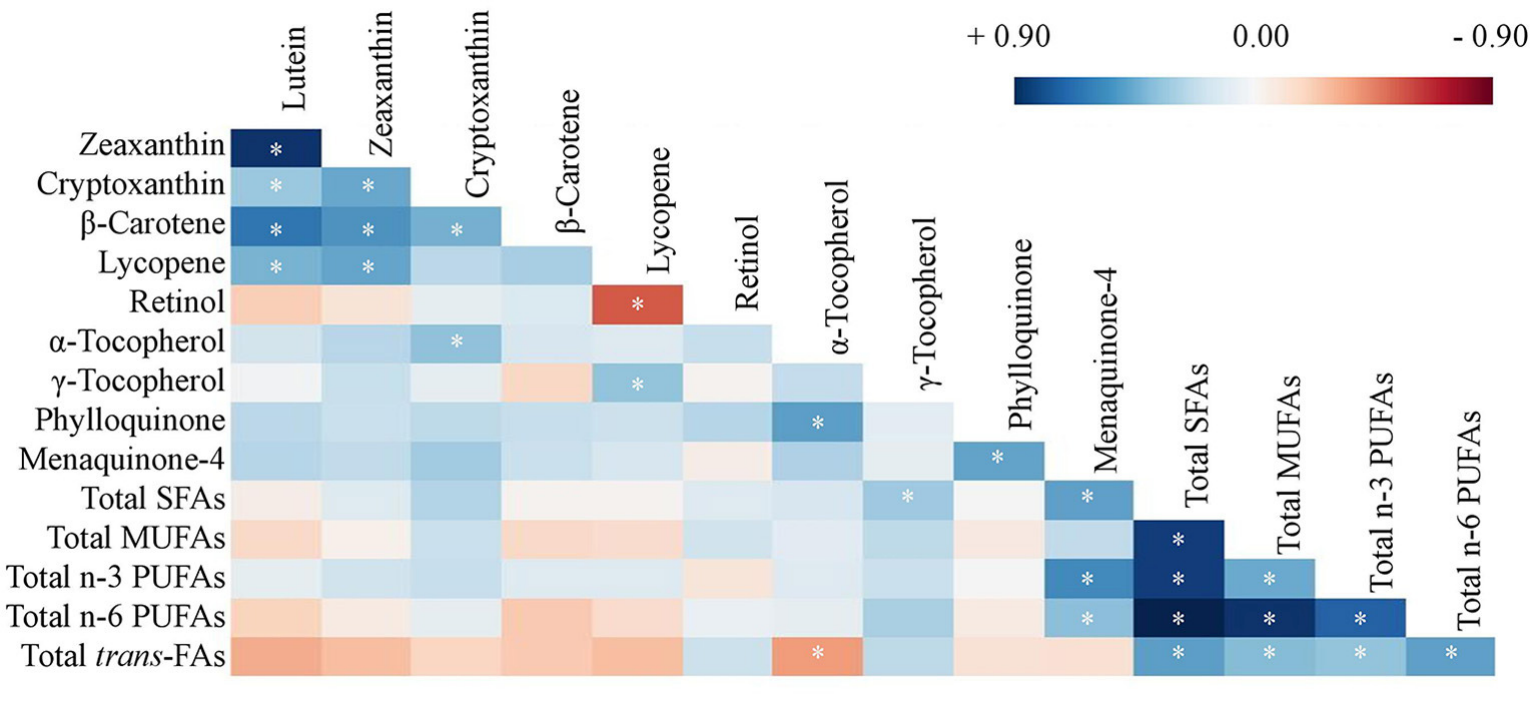
Heat map of Pearson's correlation coefficients of concentrations of carotenoids, retinol, tocopherols, phylloquinone, menaquinone-4, and fatty acids averaged from frontal and temporal cortices (except vitamin K only in the frontal cortex, *n* = 47, *p < 0.05). Log transformation has been applied to all nutrient concentrations. SFA, saturated fatty acid; MUFA, monounsaturated fatty acid; PUFA, polyunsaturated fatty acid; *trans*-FA, *trans*-fatty acid.

As shown in Figure 1, significant correlations were identified among concentrations of carotenoids, and among FAs (blue represents positive and red represents negative correlations). For others that also reached statistical significance, lycopene was also positively correlated with γ-TP (*r* = 0.32, *p* = 0.027) and negatively correlated with vitamin A (*r* = −0.49, *p* < 0.001), while α-TP was positively correlated with PK (*r* = 0.46, *p* = 0.002) and negatively associated with total *trans*-FAs (*r* = −0.32, *p* = 0.030). MK-4, but not PK, was also associated with FA concentrations including total SFA (*r* = 0.45, *p* = 0.002), n-3 PUFA (*r* = 0.52, *p* < 0.001), and n-6 PUFA (*r* = 0.34, *p* = 0.023). These correlations among nutrient concentrations further warranted the investigation of nutrients as NPs.

Next, PCA was used to derive NPs from brain nutrient concentrations. The first five NPs that explain the highest variance (each of which had an eigenvalue >1.0) among the 47 subjects are described in Table 3. No obvious outlier was detected in a PCA plot (data not shown). NP1 which has the highest variance accounted for 26.20% of the total variance. NP1 is described as higher concentrations of SFAs, MUFAs, and n-3 and n-6 PUFAs (all loading coefficients >0.40). NP2 is described by high concentrations of carotenoids (all loading coefficients ≥0.30), and NP3 is described by high concentrations of retinol, α-TP, and PK (all loading coefficients ≥0.40). The correlations of these nutrients in each NP are as depicted in Figure 1. The first five NPs accounted for 75.92% of the total variance of the original nutrient dataset.

**Table 3:** Construction of nutrient patterns (NPs), NP structure and variance explained (*n* = 47).

**Nutrient**	**NP1**	**NP2**	**NP3**	**NP4**	**NP5**
**Carotenoids**					
Lutein	0.02	0.45	−0.14	0.23	0.12
Zeaxanthin	0.09	0.44	−0.13	0.14	0.27
Cryptoxanthin	0.16	0.30	0.14	0.11	0.06
β-Carotene	0.01	0.37	0.08	0.43	0.11
Lycopene	0.02	0.32	−0.41	−0.31	0.03
Retinol (Vitamin A)	0.06	−0.09	0.61	0.18	0.40
**Vitamin E**					
α-TP	0.11	0.23	0.40	−0.42	−0.04
γ-TP	0.19	0.05	−0.16	−0.54	0.52
**Vitamin K**					
PK	0.07	0.23	0.41	−0.28	−0.11
MK-4	0.26	0.19	0.12	−0.04	−0.50
**Fatty acids**					
Total SFAs	0.50	−0.05	−0.05	0.07	−0.03
Total MUFAs	0.41	−0.13	0.02	0.04	0.08
Total n-3 PUFAs	0.41	0.03	−0.13	0.12	−0.23
Total n-6 PUFAs	0.46	−0.15	−0.06	0.01	−0.05
Total *trans*–FAs	0.22	−0.27	−0.08	0.15	0.37
**Eigenvalue**	3.89	3.39	1.68	1.27	1.06
**% Variance**	26.2	22.76	11.28	8.53	7.15
**Cumulative % variance**	26.2	48.96	60.24	68.77	75.92

*SFA, saturated fatty acid; MUFA, monounsaturated fatty acid; PUFA, polyunsaturated fatty acid; trans-FA, trans-fatty acid; TP, tocopherol; PK, phylloquinone; MK-4, menaquinone-4*.

### Composite Scores on Cognitive Domains, Depression, and Activities of Daily Living

The time interval between the cognitive assessment at the time point closest to death and the autopsy was <1 year for all subjects with an average of 156 (93) days for those whose data could be accurately calculated (81%). Subjects with GDS 1–3 had significantly higher MMSE scores than those with GDS 4–7 (*p* < 0.001). Similarly, SIB score was higher in subjects with GDS 1–3 (*p* < 0.001). Therefore, GDS effectively separated subjects based on their performance on global cognition as also previously reported in the original GCS cohort (*n* = 244) (56). Further, the composite scores for cognitive domains, depression, and activities of daily living had been calculated (Supplementary Table 3). Subjects with GDS 1–3 had significantly higher composite scores on all six cognitive domains and activities of daily living, and significantly lower scores on depression (less depression). Of note, while composite scores for other cognitive domains were available for all subjects, visuospatial function score was available for 87% of participants without dementia and 50% of participants with dementia, and attention score was available for 70% of participants without demented and 46% of participants with dementia.

### Relationship Between Brain Nutrient Concentrations and Cognition

Scores of the first five NPs were not statistically different between demented and non-demented subjects (data not shown). However, among non-demented subjects, subjects who had mild cognitive impairment (GDS 3, *n* = 11) had significantly lower NP2 score than that of cognitively intact subjects (GDS 1–2, *n* = 12, *p* = 0.002), but not for other NPs. The difference remained statistically significant after an adjustment for sex, education, hypertension, diabetes, and presence of *APOE* **ε**4 allele (*p* = 0.004).

**Figure 2:**
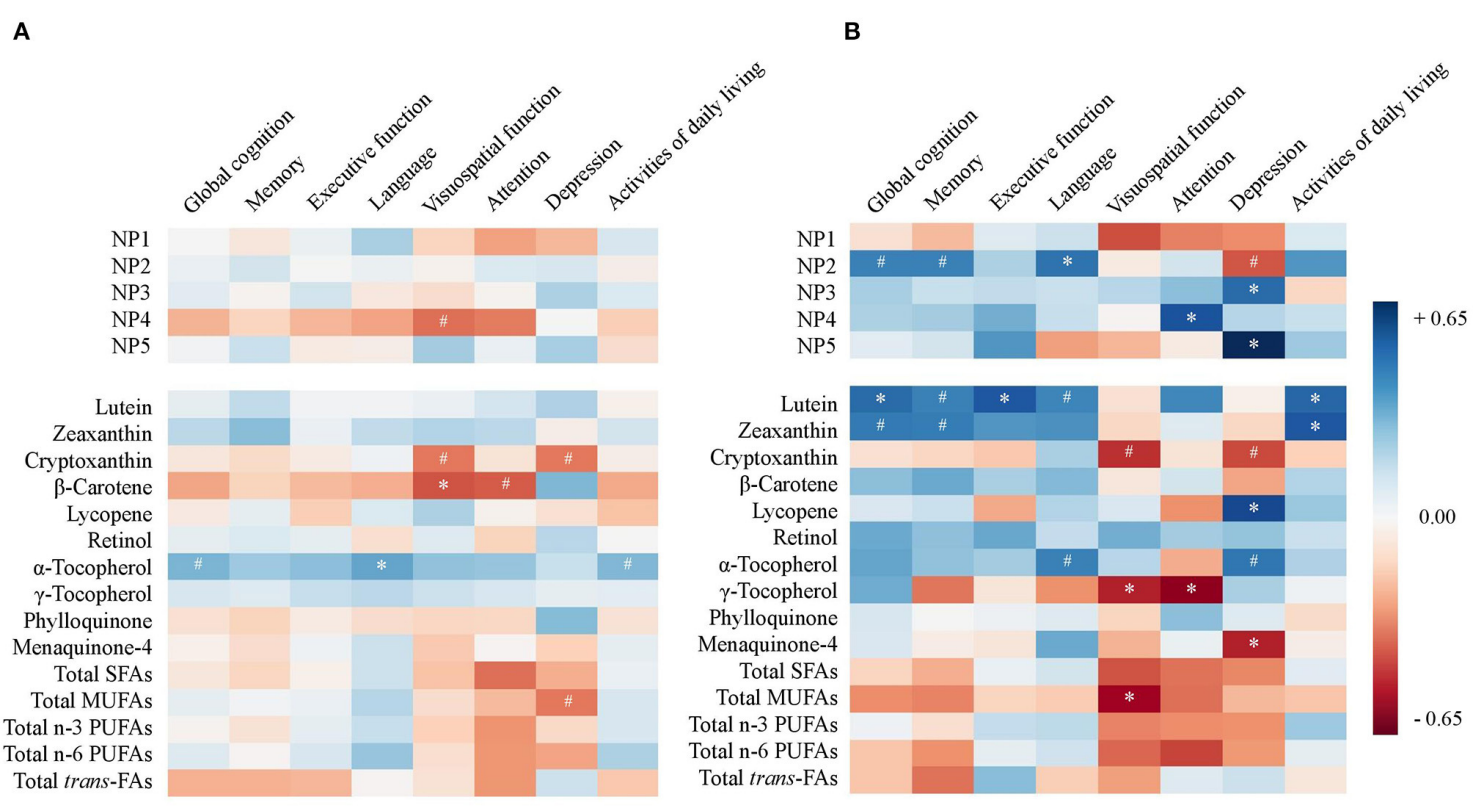
Heat map of Pearson's correlation coefficients between nutrient pattern (NP) scores or nutrient concentrations and scores on cognitive domains, depression, and activities of daily livings in **(A)** all subjects (*n* = 47) and **(B)** non-demented subjects (Global Deterioration Scale = 1–3, *n* = 23). Correlations are adjusted for sex, education, diabetes, hypertension, and APOE ε4 allele (^#^*p* < 0.10, *p < 0.05). SFA, saturated fatty acid; MUFA, monounsaturated fatty acid; PUFA, polyunsaturated fatty acid; *trans*-FA, *trans*-fatty acid.

A heat map was constructed to provide Pearson's partial correlation coefficients between NP scores or nutrient concentrations and scores on cognitive domains, depression, and activities of daily living in all subjects (Figure 2A, *p*-values provided in Supplementary Table 4A). Pearson's partial correlations were adjusted for sex, education, diabetes, hypertension, and presence of *APOE* **ε**4 allele. No consistent relationship between NPs and cognitive domain scores that reached statistical significance was observed.

**Figure 3:**
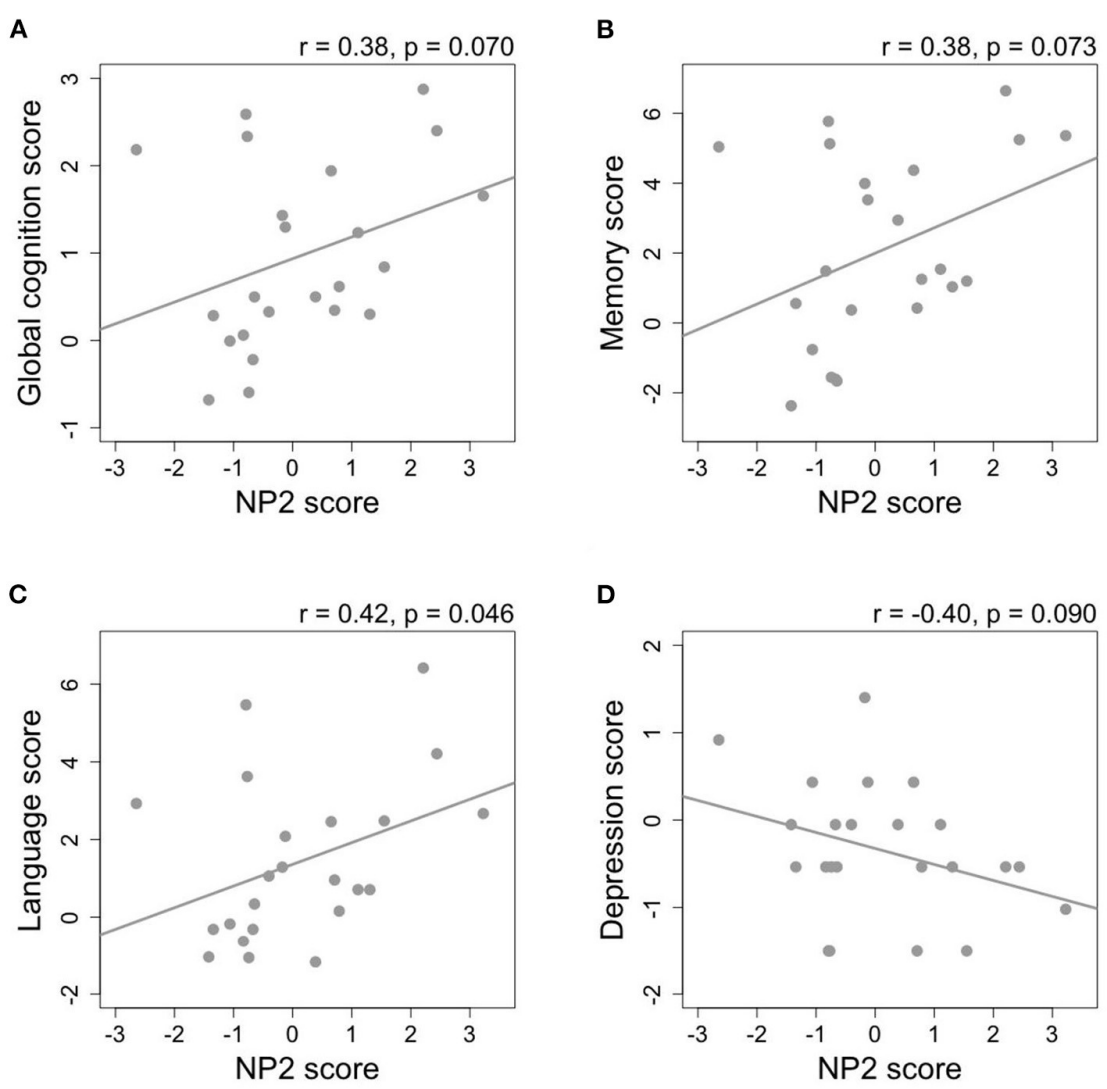
The relationship between nutrient pattern 2 (NP2) score and composite scores of **(A)** global cognition, **(B)** memory, **(C)** language, and **(D)** depression among non-demented subjects (Global Deterioration Scale = 1–3, *n* = 23). Pearson's correlation coefficients are adjusted for sex, education, diabetes, hypertension, and APOE ε4 allele.

A subset analysis among non-demented subjects (GDS 1–3) was performed and Pearson's coefficients are illustrated in a heat map (Figure 2B, *p*-values provided in Supplementary Table 4B). In the models adjusted for covariates among the five NPs representing the highest variances, NP2 was consistently associated with higher scores on global cognition (*r* = 0.38, *p* = 0.070), memory (*r* = 0.38, *p* = 0.073), language (*r* = 0.42, *p* = 0.046), and lower depression score (*r* = −0.40, *p* = 0.090) (Figure 3). After additional adjustment for antidepressant use, the correlation with lower depression score remained borderline significant (*r* = −0.35, *p* = 0.100). Since NP2 is mainly described by carotenoids, significant correlations were also consistently observed between lutein, zeaxanthin, β-carotene and scores on global cognition, memory, language, and depression. Other notable associations included NP3 and NP5 and higher depression. Additional adjustment for antithrombotic use was performed for PK and MK-4 and their correlations with different cognitive domain composite scores remained statistically non-significant (*p* > 0.05) for all six cognitive domains and activities of daily living, while the correlation between MK-4, but not PK, and depression remained statistically significant (Supplementary Table 4B).

## Discussion

This study documents that brain NP high in carotenoids was consistently associated with better performance on multiple cognitive domains, activities of daily living, and lower depression among non-demented older adults in the GCS. Results also confirm previously established positive relationships between serum and brain concentrations of carotenoids in this group of subjects independent of their cognitive status (27). Given that serum concentrations of carotenoids likely reflect their habitual intake in the oldest old as previously discussed (27), our findings in the present study underscore the timing of intervention with diet high in carotenoid content before the onset of age-related dementia. This is further supported by the fact that nutrient concentrations and NP scores were not different between demented and non-demented subjects. Our exploratory findings also corroborate previous findings where serum levels of carotenoids are positively associated with better cognition in aging subjects (26, 32, 57–60). Specifically, higher serum lutein concentration was reported to be associated with better performance on language (32), which is similar to the correlation between NP2 and language score in this study.

The present analysis investigated concentrations of nutrients in the brain, the organ most relevant to cognition, as compared to previous studies that have established dietary or serum NPs with similar exploratory approaches (10, 11, 13, 15). Although the relationship between better adherence to *a priori* hypothesized intake patterns (such as MeDi, HDI, HEI, DASH, MIND) and lower risk of cognitive decline have been established (2), *a priori* hypothesized NPs are difficult with brain concentrations since little is known regarding nutrient uptake across the BBB and nutrient metabolism in neural tissue. For instance, SFAs and MUFAs can be *de novo* synthesized in the liver and CNS and may not reflect intake levels (61), and among n-3 PUFAs, DHA preferentially accumulates in neural tissue (62, 63). While substitution of SFA and *trans*-FA intakes with MUFAs and PUFAs decreases risk of age-related cognitive impairment in many prospective cohorts (64), our findings with brain content cannot be directly compared with intake levels of SFAs and MUFAs. It has also been reported that higher SFA content in membranes is usually associated with higher PUFA content to maintain membrane stability (65, 66), which likely explains the high correlation between SFA and unsaturated FA observed in the GCS brain tissues. Similarly, retinol is thought to be either taken up into the brain by STRA6, a retinol-binding protein-receptor detected at the BBB or derived directly from the cleavage of provitamin A carotenoids (β-carotene and β-cryptoxanthin) by the enzyme BCO1 detected in human brain (67, 68). It remains unknown how much each source contributes to vitamin A content in the brain. Moreover, a previous report on vitamin K metabolism in rat cerebellum also suggests that neural MK-4 content is regulated by the enzyme UBIAD1 (69). Overall, findings of nutrient levels in neural tissue need to be cautiously interpreted for dietary recommendations, especially for nutrients that can be derived from other substrates and nutrients whose levels are tightly regulated in the brain. Data on dietary intake in the GCS have been previously reported (70). However, the dietary assessment was subjective and might have overestimated or underestimated food intake, particularly in this population with varying degrees of cognitive performance. As a result, dietary intake data were not incorporated into the present analysis.

Age-related cognitive impairment, notwithstanding mixed clinical pathologies, shares molecular signatures of increased oxidative stress and neuroinflammation (71, 72). Both carotenoids and n-3 PUFAs, especially lutein and DHA both of which are selectively accumulated in the brain, have been proposed to interfere with the progression of cognitive impairment in aging, presumably owing to their antioxidative and anti-inflammatory properties (73, 74). Consistent with previous studies investigating neural concentrations of individual nutrients, a significant relationship was observed with lower carotenoids (mainly lutein and zeaxanthin) among cognitively impaired or demented subjects (26, 75–78). However, as previously discussed by Zamroziewicz and Barbey (22), univariate analytical approach with individual nutrients may be confounded by the effect of NPs and does not address the potentially interactive effects of multiple nutrients on cognitive health. An exploratory trial demonstrates that a combination of lutein and DHA supplements statistically improved performance on memory and learning in cognitively unimpaired elder women after 4 months whereas lutein or DHA supplement alone did not (79). Moreover, most individuals predominantly acquire nutrients from dietary sources that consist of a complex combination of nutrients. While a 6-month intervention with a lutein and zeaxanthin supplement failed to improve cognitive outcomes in subjects with or without Alzheimer's disease (80), a daily intervention with an avocado (a highly bioavailable source of 0.5 mg lutein and zeaxanthin, along with being a good source of potassium, B vitamins, vitamins C, E, K, MUFAs, and other non-essential phytochemicals) for 6 months has shown to improve cognitive performance on the Spatial Working Memory and the Stockings of Cambridge in non-demented subjects with low baseline intake of lutein-rich foods (81, 82). In the present analysis, a multivariate analysis approach (PCA) has been adopted to address correlations among nutrients and inspect the nutrition variable as NPs which reflect how multiple nutrients may synergistically function in the context of cognitive functioning and age-related cognitive impairment. This is an important step in the field of nutritional cognitive neuroscience toward the application of emerging technologies (such as metabolomics and brain magnetic resonance imaging) to systematically identify underlying mechanisms that mediate the effect that a combination of nutrients have on clinical outcomes (22, 32).

We acknowledge that this exploratory study is limited by a relatively small sample size (which is reflected by borderline significant *p*-values in Supplementary Table 4B) of mostly Caucasian women, and the inability to control for other covariates that may affect cognitive function such as alcohol and smoking history, physical activity, social interactions, and genetics (83). However, nutrient profiles and concentrations in this current analysis are similar to those of other cohorts of older adults (29–31, 84–88). NP1, described mostly by high fat content, was not associated with cognition in this population. Previous studies have reported benefits of diets high in n-3 PUFAs, especially DHA, on cognitive health (33, 89). While it is more appropriate to use absolute concentrations of FAs in the PCA, relative concentrations of FAs (i.e., FA composition) may be more relevant to the biological function of the brain. Additionally, it is unclear if the FA compositions in the brain of this cohort of the oldest old were in the normal range, since altered fatty acid compositions among cognitively impaired or demented subjects were reported (29–31), but no difference were observed between those with and without dementia in this study.

Other nutrients and dietary compounds such as B vitamins, vitamin D, minerals, and polyphenols that may be beneficial to cognitive health were also not examined in this study (90), but propose the opportunity to expand the scope of investigation. Nutrients that are not present in the brain but sharing common dietary sources with carotenoids and n-3 PUFAs, such as fibers in fruits, vegetables, nuts and seeds, may provide additional benefits to the central nervous system by functioning systemically through the regulation of reverse cholesterol transport, gut microbiota, and gut-brain axis signaling (91, 92). Finally, a cross-sectional study does not address a causal and longitudinal relationship between nutrition and cognition. A reverse causation where cognitive impairment leads to changes in nutrient uptake and metabolism—for example through BBB breakdown—is possible (23). However, dietary intervention in human trials and animal studies have indicated a significant impact that nutrition has on cognitive health in aging (93–95).

In summary, this report is the first to adopt a multivariate analysis approach to address the co-existence of nutrients and dietary compounds in the brain when investigating the relationship between nutrition and cognitive function in an aging population. Our findings support beneficial effects of a NP higher in carotenoids potentially derived from a diet rich in fruits and vegetables similar to the MeDi and DASH diets, on lowering the risk of age-related cognitive impairment and dementia previously reported (2, 93, 94). As compared to symptoms of nutritional deficiency which could be caused by an inadequate intake of one single nutrient and manifest within a short period of time, we are aware of the need to assess diet as a dietary pattern or NP in a context of complex outcomes such as age-related cognitive impairment (20–22). The synergistic and cumulative effect of nutrients on a person's risk of chronic diseases have recently been highlighted in the Dietary Guidelines for Americans 2015–2020 and 2020–2025 (96, 97).

## Data Availability Statement

The data analyzed in this study is subject to the following licenses/restrictions: The data set is not publicly available but it is available on request from the corresponding author, Elizabeth J. Johnson. Requests to access these datasets should be directed to elizabeth.johnson@tufts.edu.

## Ethics Statement

The studies involving human participants were reviewed and approved by the University of Georgia Institutional Review Board. Separate approval for using de-identified data for the present analyses was obtained from the Tufts University/Tufts Medical Center Institutional Review Board. The patients/participants provided their written informed consent to participate in this study.

## Author Contributions

JT, TMS, MAJ, LWP, and EJJ designed the study. MAJ and LWP contributed to the original design of the GCS and collection of biological samples. RV analyzed carotenoid, vitamin A, and vitamin E concentrations in all samples. NRM and AHL analyzed fatty acid concentration in all samples. GF analyzed vitamin K concentrations in all samples. JT performed statistical analysis, wrote the paper, and had primary responsibility for final content. All authors interpreted the data, contributed to the article, and approved the submitted version.

## Conflict of Interest

The authors declare that the research was conducted in the absence of any commercial or financial relationships that could be construed as a potential conflict of interest.
