# Supplementary material for: Carotenoid-Rich Brain Nutrient Pattern Is Positively Correlated With Higher Cognition and Lower Depression in the Oldest Old With No Dementia
Source: Front Nutr. 2021 Jun 29;8:704691. doi: 10.3389/fnut.2021.704691 (PMC8275828; doi:10.3389/fnut.2021.704691)
Supplement: Supplementary file 1 [file Table_1.docx]

**Supplementary Table 1** Calculation of composite scores of cognitive domains, depression, and activities of daily living (adapted from Bowman et al (14))

| **Composite score** | **Cognitive tests/domains used** |
| --- | --- |
|  |  |
| Global cognition  Memory  Executive function  Language  Visuospatial function  Attention  Depression  Activities of daily living | MMSE, SIB, Memory score, Executive function score, Language score, Visuospatial function score, Attention score  FOME recall, FOME retention, CP recall, WLMT recognition  BDS, WAIS-III Similarities  BNT, VF, COWAT  CP total  WLMT list learning  GDSSF  DAFS |

MMSE: Mini-Mental State Examination, SIB: Severe Impairment Battery, FOME: Fuld Object Memory Evaluation, CP: Constructional Praxis, WLMT: Word List Memory Test, BDS: Behavioral Dyscontrol Scale, WAIS: Wechsler Adult Intelligence Scale, BNT: Boston Naming Test, VF: Verbal Fluency, COWAT: Controlled Oral Word Association Test, GDSSF: Geriatric Depression Scale-Short Form, DAFS: Direct Assessment of Functional Status
