# Supplementary material for: Carotenoid-Rich Brain Nutrient Pattern Is Positively Correlated With Higher Cognition and Lower Depression in the Oldest Old With No Dementia
Source: Front Nutr. 2021 Jun 29;8:704691. doi: 10.3389/fnut.2021.704691 (PMC8275828; doi:10.3389/fnut.2021.704691)
Supplement: Supplementary file 2 [file Table_2.docx]

**Supplementary Table 2** Mean (SD) of individual fatty acid (FAs) concentrations averaged from the frontal and temporal cortices in 47 centenarians

| **FA** | **Absolute concentration**  **(nmol/mg)** | **Relative concentration**  **(mol%)** |
| --- | --- | --- |
| Saturated FA  10:0  12:0  14:0  15:0  16:0  18:0  20:0  22:0  24:0  Monounsaturated FA  16:1, n-9  16:1, n-7  18:1, n-9  18:1, n-7  20:1, n-9  22:1, n-9  24:1, n-9  n-3 Polyunsaturated FA  18:3, n-3  18:4, n-3  20:3, n-3  20:5, n-3  22:5, n-3  22:6, n-3  n-6 Polyunsaturated FA  18:2, n-6  18:3, n-6  20:2, n-6  20:3, n-6  20:4, n-6  22:2, n-6  22:4, n-6  22:5, n-6  *trans*-FA  16:1, n-9  16:1, n-7  18:1, n-10 to n-12  18:1, n-9  18:1, n-7  18:2 TT/TCTX  18:2 CLA | 4.73E-3 (1.54E-3)  2.83E-2 (1.34E-2)  1.40E-1 (3.12E-2)  4.55E-2 (9.12E-2)  8.02 (1.23)  7.04 (1.02)  4.29E-2 (9.38E-3)  1.97E-2 (1.66E-2)  3.02E-2 (2.12E-2)  1.59E-1 (3.64E-2)  2.28E-1 (1.19E-1)  5.22 (1.91)  1.17 (0.28)  1.58E-1 (8.58E-2)  1.58E-2 (5.05E-3)  8.40E-2 (6.47E-2)  6.83E-2 (3.97E-2)  6.31E-2 (3.97E-2)  1.81E-3 (9.34E-4)  1.05E-1 (7.19E-2)  1.99E-1 (5.73E-2)  3.77 (0.65)  6.54E-1 (6.20E-1)  8.49E-3 (3.76E-3)  4.26E-2 (1.98E-2)  2.88E-1 (7.00E-2)  2.72 (0.38)  8.94E-3 (6.75E-3)  1.36 (0.28)  4.28E-1 (1.96E-1)  5.13E-2 (1.60E-2)  2.34E-2 (9.00E-3)  6.85E-3 (8.17E-3)  7.61E-2 (3.10E-2)  4.27E-2 (2.22E-2)  3.313E-2 (1.16E-2)  2.06E-2 (9.69E-3) | 0.02 (0.01)  0.09 (0.03)  0.45 (0.09)  0.16 (0.33)  25.02 (1.00)  21.92 (1.18)  0.13 (0.02)  0.06 (0.04)  0.09 (0.05)  0.49 (0.06)  0.68 (0.18)  15.54 (2.16)  3.59 (0.44)  0.45 (0.16)  0.05 (0.01)  0.23 (0.14)  0.19 (0.07)  0.18 (0.09)  0.006 (0.003)  0.30 (0.16)  0.61 (0.15)  11.90 (1.60)  1.94 (1.11)  0.03 (0.01)  0.13 (0.05)  0.88 (0.15)  8.54 (0.57)  0.02 (0.01)  4.17 (0.38)  1.34 (0.44)  0.16 (0.05)  0.07 (0.02)  0.02 (0.02)  0.24 (0.08)  0.14 (0.07)  0.10 (0.03)  0.06 (0.03) |

18:2 TT/TCTX: *trans*-9, *trans*-12-octadecenoic acid, 18:2 CLA: conjugated linoleic acid.
