# Supplementary material for: Carotenoid-Rich Brain Nutrient Pattern Is Positively Correlated With Higher Cognition and Lower Depression in the Oldest Old With No Dementia
Source: Front Nutr. 2021 Jun 29;8:704691. doi: 10.3389/fnut.2021.704691 (PMC8275828; doi:10.3389/fnut.2021.704691)
Supplement: Supplementary file 3 [file Table_3.docx]

**Supplementary Table 3** Mean (SD) of composite scores of cognitive domains, depression, and activities of daily living

| **Cognitive domain** | **GDS 1-3**  **(n = 23)** | **GDS 4-7**  **(n = 24)** | **p value ^a^** |
| --- | --- | --- | --- |
| Global cognition  Memory  Executive function  Language  Visuospatial function ^b^  Attention ^b^  Depression  Activities of daily living | 0.96 (1.05)  2.08 (2.74)  1.19 (1.36)  1.43 (2.09)  0.46 (0.86)  0.59 (0.87)  -0.35 (0.77)  0.60 (0.65) | -1.03 (1.07)  -1.99 (1.89)  -1.14 (1.45)  -1.37 (1.74)  -0.76 (0.72)  -0.85 (0.31)  0.44 (1.10)  -0.58 (0.94) | < 0.001  < 0.001  < 0.001  < 0.001  < 0.001  < 0.001  0.015  < 0.001 |

GDS: Global Deterioration Scale

^a^ Comparisons between GDS 1-3 and GDS 4-7 using Student’s t-test.

^b^ Visuospatial function score was available for 20 nondemented (87%) and 12 demented subjects (50%), and attention score was available for 16 nondemented (70%) and 11 demented subjects (46%).
