# Supplementary material for: Carotenoid-Rich Brain Nutrient Pattern Is Positively Correlated With Higher Cognition and Lower Depression in the Oldest Old With No Dementia
Source: Front Nutr. 2021 Jun 29;8:704691. doi: 10.3389/fnut.2021.704691 (PMC8275828; doi:10.3389/fnut.2021.704691)
Supplement: Supplementary file 4 [file Table_4.docx]

**Supplementary Table 4** P values for Pearson’s correlation test between nutrient concentrations averaged from frontal and temporal cortices and cognitive domain scores in (**A**) all subjects (n = 47) and (**B**) nondemented subjects (Global Deterioration Scale or GDS = 1-3, n = 23).

**Supplementary Table 4A**

| **All subjects (n = 47)** | **Global cognition** | **Memory** | **Executive function** | **Language** | **Visuospatial function** | **Attention** | **Depression** | **Activities of daily living** |
| --- | --- | --- | --- | --- | --- | --- | --- | --- |
| NP1  NP2  NP3  NP4  NP5  Lutein  Zeaxanthin  Cryptoxanthin  β-Carotene  Lycopene  Retinol  α-TP  γ-TP  PK  MK-4  SFAs  MUFAs  n-3 PUFAs  n-6 PUFAs  *trans*-FAs | 0.980  0.826  0.727  0.204  0.948  0.774  0.315  0.727  0.143  0.764  0.787  0.070  0.581  0.625  0.892  0.712  0.793  0.944  0.674  0.192 | 0.716  0.532  0.942  0.452  0.427  0.355  0.105  0.519  0.419  0.761  0.642  0.176  0.659  0.439  0.566  0.460  0.929  0.643  0.963  0.189 | 0.853  0.979  0.523  0.213  0.808  0.952  0.829  0.806  0.237  0.368  0.791  0.108  0.389  0.814  0.906  0.872  0.824  0.806  0.600  0.208 | 0.222  0.857  0.753  0.136  0.863  0.941  0.349  0.900  0.174  0.637  0.609  0.038  0.316  0.594  0.464  0.452  0.287  0.392  0.143  0.953 | 0.526  0.929  0.642  0.079  0.293  0.864  0.363  0.091  0.038  0.339  0.727  0.209  0.541  0.553  0.440  0.382  0.651  0.483  0.659  0.718 | 0.255  0.737  0.955  0.130  0.878  0.655  0.454  0.757  0.073  0.924  0.552  0.277  0.697  0.622  0.967  0.109  0.379  0.200  0.205  0.204 | 0.260  0.612  0.274  0.983  0.243  0.285  0.854  0.055  0.101  0.654  0.334  0.445  0.793  0.130  0.442  0.203  0.054  0.528  0.164  0.504 | 0.566  0.842  0.642  0.367  0.566  0.866  0.526  0.862  0.150  0.284  0.976  0.085  0.735  0.664  0.801  0.824  0.575  0.576  0.234  0.323 |

**Supplementary Table 4B**

| GDS 1-3 (n = 23) | Global cognition | Memory | Executive function | Language | Visuospatial function | Attention | Depression | Activities of daily living |
| --- | --- | --- | --- | --- | --- | --- | --- | --- |
| NP1  NP2  NP3  NP4  NP5  Lutein  Zeaxanthin  Cryptoxanthin  β-Carotene  Lycopene  Retinol  α-TP  γ-TP  PK  MK-4  SFAs  MUFAs  n-3 PUFAs  n-6 PUFAs  *trans*-FAs | 0.733  0.070  0.403  0.422  0.816  0.035  0.059  0.736  0.278  0.733  0.175  0.163  0.181  0.711  0.747  0.602  0.215  0.942  0.478  0.481 | 0.427  0.073  0.572  0.389  0.671  0.072  0.062  0.613  0.177  0.585  0.291  0.304  0.152  0.987  0.892  0.354  0.195  0.720  0.227  0.149 | 0.778  0.422  0.523  0.192  0.114  0.016  0.118  0.499  0.411  0.332  0.166  0.386  0.789  0.913  0.804  0.889  0.609  0.547  0.871  0.269 | 0.598  0.046  0.560  0.552  0.287  0.078  0.106  0.407  0.243  0.451  0.546  0.070  0.228  0.777  0.203  0.672  0.526  0.509  0.639  0.542 | 0.102  0.876  0.515  0.980  0.431  0.759  0.659  0.056  0.826  0.757  0.231  0.518  0.039  0.644  0.446  0.111  0.020  0.232  0.149  0.331 | 0.286  0.726  0.377  0.042  0.891  0.158  0.832  0.816  0.731  0.329  0.480  0.444  0.029  0.400  0.925  0.242  0.235  0.317  0.126  0.823 | 0.215  0.090  0.035  0.472  0.001  0.916  0.643  0.065  0.327  0.008  0.311  0.054  0.407  0.787  0.035  0.201  0.417  0.228  0.258  0.601 | 0.759  0.117  0.639  0.569  0.360  0.028  0.016  0.550  0.456  0.347  0.588  0.438  0.942  0.671  0.909  0.808  0.479  0.353  0.871  0.801 |

NP: nutrient pattern, TP: tocopherol, PK: phylloquinone, MK-4: menaquinone, SFAs: saturated fatty acids, MUFAs: monounsaturated fatty acids, PUFAs: polyunsaturated fatty acids, *trans*-FAs: *trans*-fatty acids
